# Effect of different parameters utilized for image guided endodontic root canal preparation on temperature changes: an in vitro study

**DOI:** 10.1186/s12903-023-03799-x

**Published:** 2024-01-13

**Authors:** Zsolt Rajnics, Iván Mandel, Ákos Nagy, Kinga Turzó, Attila Mühl, Gyula Marada

**Affiliations:** Dental School, Medical Faculty, University of Pécs, Tüzér u. 1, Pécs, H-7623 Hungary

**Keywords:** Guided endodontics, Access cavity, Temperature, Root canal

## Abstract

**Background:**

Navigated endodontics is a cutting-edge technology becoming increasingly more accessible for dental practitioners. Therefore, it is necessary to clarify the ideal technical parameters of this procedure to prevent collateral damage of the surrounding tissues. There is a limited number of studies available in published scientific literature referencing the possible collateral thermal damage due to high-speed rotary instruments used in guided endodontic drilling. The aim of our study was to investigate the different drilling parameters and their effect upon the temperature elevations measured on the outer surface of teeth during guided endodontic drilling.

**Methods:**

In our in vitro study, 72 teeth with presumably narrow root canals were prepared using a guided endodontic approach through a 3D-printed guide. Teeth were randomly allocated into six different test groups consisting of 12 teeth each, of which, four parameters affecting temperature change were investigated: (a) access cavity preparation prior to endodontic drilling, (b) drill speed, (c) cooling, and (d) cooling fluid temperature. Temperature changes were recorded using a contact thermocouple electrode connected to a digital thermometer.

**Results:**

The highest temperature elevations (14.62 °C ± 0.60 at 800 rpm and 13.76 °C ± 1.24 at 1000 rpm) were recorded in the groups in which drilling was performed without prior access cavity preparation nor without a significant difference between the different drill speeds (p = 0.243). Access cavity preparation significantly decreased temperature elevations (p < 0.01) while drilling at 800 rpm (8.90 °C ± 0.50) produced significantly less heating of the root surface (p < 0.05) than drilling at 1000 rpm (10.09 °C ± 1.32). Cooling significantly decreased (p < 0.01) temperature elevations at a drill speed of 1000 rpm, and cooling liquid temperatures of 4–6 °C proved significantly (p < 0.01) more beneficial in decreasing temperature elevations (1.60 °C ± 1.17) than when compared with room temperature (21 °C) liquids (4.01 °C ± 0.22).

**Conclusions:**

Based on the results of our study, guided endodontic drilling at drill speeds not exceeding 1000 rpm following access cavity preparation, with constant cooling using a fluid cooler than room temperature, provides the best results in avoiding collateral thermal damage during navigated endodontic drilling of root canals.

## Introduction

Preparation of dental hard tissues using high-speed rotary instruments generates heat; therefore, adequate cooling of the preparation area must be provided to prevent collateral thermal damage of the surrounding tissues [1]. Heat generation and cooling have been widely investigated for dental implant site preparation including the use of navigation guides. However, circumstances during navigated endodontic drilling in dentine significantly differ from navigated implant site preparation in human bone.

The most common cause of artificial root canal preparation is a narrow and/or calcified root canal; therefore, the drill encounters high resistance. This leads to increased heat generation in the case of implant site preparation in human bone, which is generally softer than dentine [2]. Although bone is not a particularly well-vascularized tissue, its blood flow may decrease collateral thermal damage contrary to dentine, which has absolutely no blood supply. In the case of bone, the thermally affected tissue is at the site of the preparation, while in the case of root preparation, the entire root membrane must be protected from the heat generated during drilling procedures [3].

The working length of endodontic drills is generally longer than those of implant drills. The efficiency of cooling decreases with a longer distance of the working end of the instrument from the cooling source and with longer preparation depths (effective working length) [4]. Cooling efficiency may be further decreased with the use of navigation guides. To overcome this negative effect, a gap between the drill guide sleeve and the gingiva is often maintained during the fabrication of dental implant surgical guides to ensure the access of the coolant to the drill [5]. However, due to the flexibility of narrower and longer drills used in endodontics, this is rarely possible during navigated endodontic drilling. Another disadvantage of drills thinner than 1.5 mm is they do not have a heat-retaining mass, and their temperature increases faster during the process of drilling.

Due to these circumstances, clinicians may expect more heat generation during guided endodontic drilling than during guided implant site preparation.

Although guided endodontic drilling is a cutting-edge technology [6], there are a limited number of reports in scientific literature referencing temperature changes during guided endodontic drilling, of which, the effect of different drilling parameters has not been investigated in detail. [7]

The aim of our study was to determine the temperature changes of root surfaces during guided endodontic drilling with various parameters. Due to the anatomical differences between natural teeth and the varying amounts of calcified dentine embedded in teeth, a large variance of results is expected.

## Materials and methods

### Sample preparation

In this study, seventy-two teeth with presumably narrow root canals were used. Navigated endodontic drilling enables straight preparation due to the relative rigidity of drills compared to conventional endodontic instruments. Therefore, only teeth bearing a straight root were selected.

Inclusion criteria:

- Tooth extracted from a patient older than 50 years of age.
- Tooth extracted due to poor periodontal prognosis.
- Straight root.

Exclusion criteria:

- Prior endodontic treatment of the tooth.
- Presence of any of the following conditions: crown restoration, caries, periapical lesions, root resorption and/or root fracture.

**Fig. 1:**
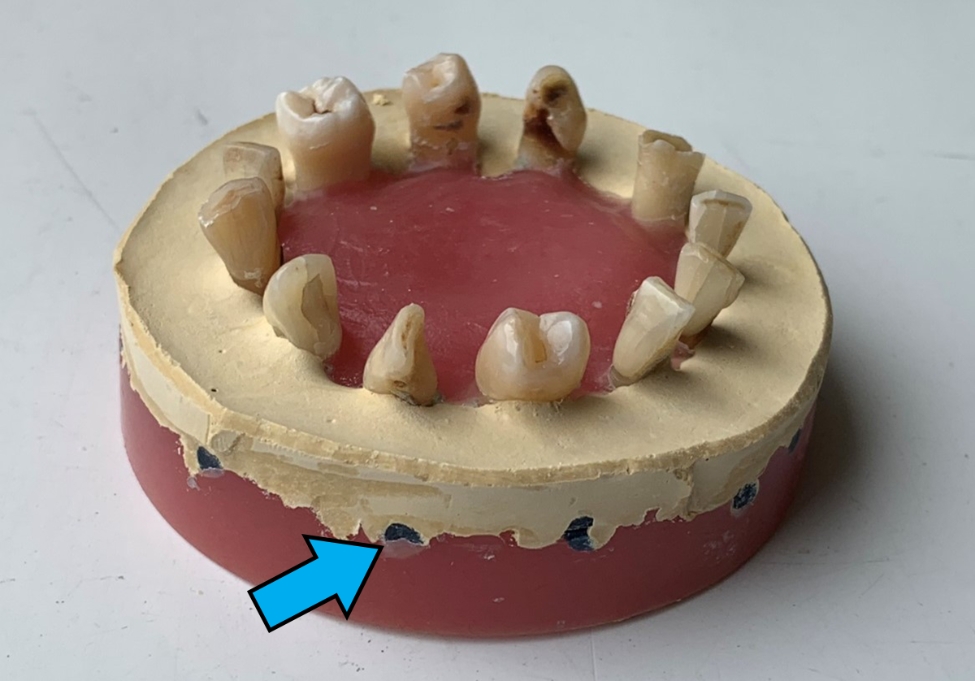
The unique stable support structure used during the study. The blue arrow indicates the external orifice of the channel for the thermocouple electrode

Root length was not standardized. However, the same effective working length was used during preparations. Variances in root canal morphology were evenly distributed among the test groups. Roots of teeth were embedded in a stable support made of plaster and acrylic resin. Each support contained twelve teeth. A channel for the thermocouple electrode was created in the support for each tooth leading to the middle of the root (Fig. 1).

**Fig. 2:**
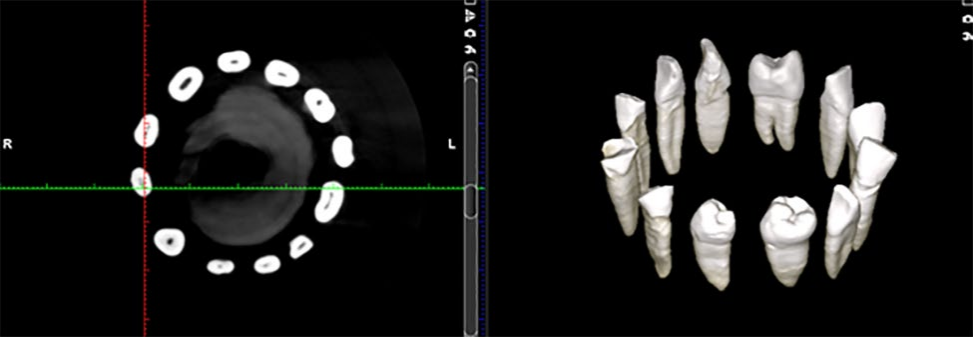
CBCT scan of the teeth inside the support structure

A CBCT scan of each support was performed utilizing the Planmeca ProMax 3D imaging system (Planmeca, Helsinki, Finland) with a resolution of 200 microns and an FOV size of 8 × 8 mm (Fig. 2).

**Fig. 3:**
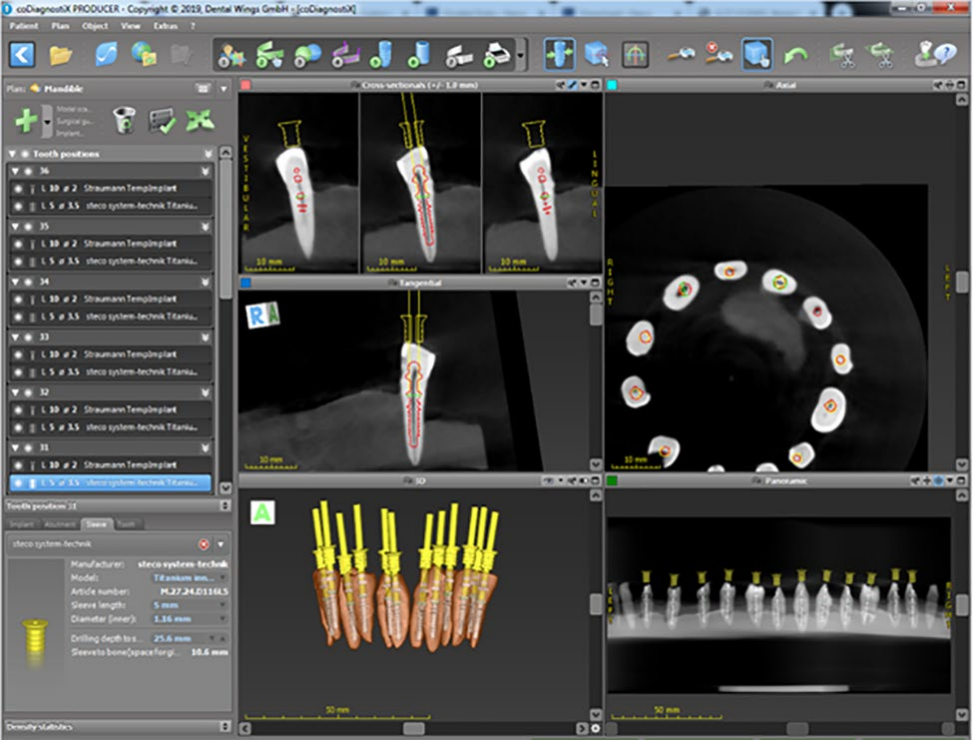
Designing the surgical template

**Fig. 4:**
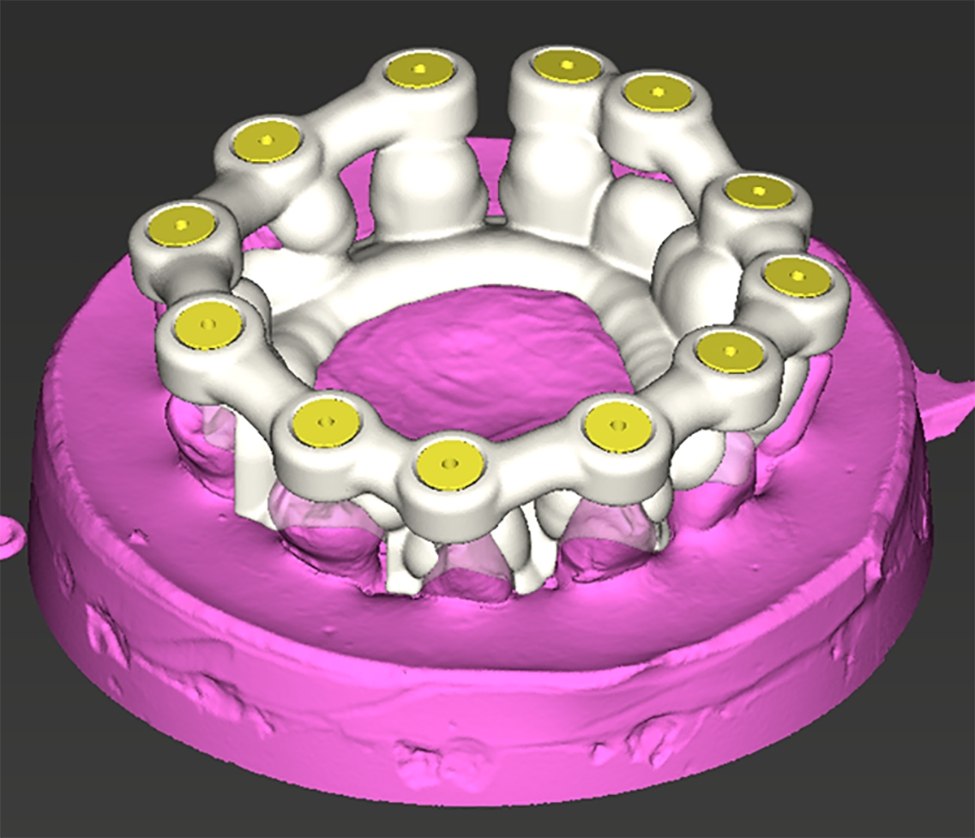
The designed surgical template

The image set was uploaded to navigated surgical planning software (coDiagnostiX - Dental Wings Inc., Montréal, Canada) (Figs. 3 and 4)).

The type of endodontic drill (1 mm diameter spiral drill - Steco-System-Technik GmbH & Co. KG, Hamburg, Germany) and the corresponding guide sleeve were selected based on the recommendation of the software manufacturer. In the design software, sleeves were positioned as close as possible to the tooth surface to minimize the effective working length. The body of the guide holding the sleeves was generated automatically by the software and 3D printed (Form2, Formlabs Inc., Somerville, USA) using clear resin (Clear Resin, Formlabs Inc., Somerville, USA).

**Fig. 5:**
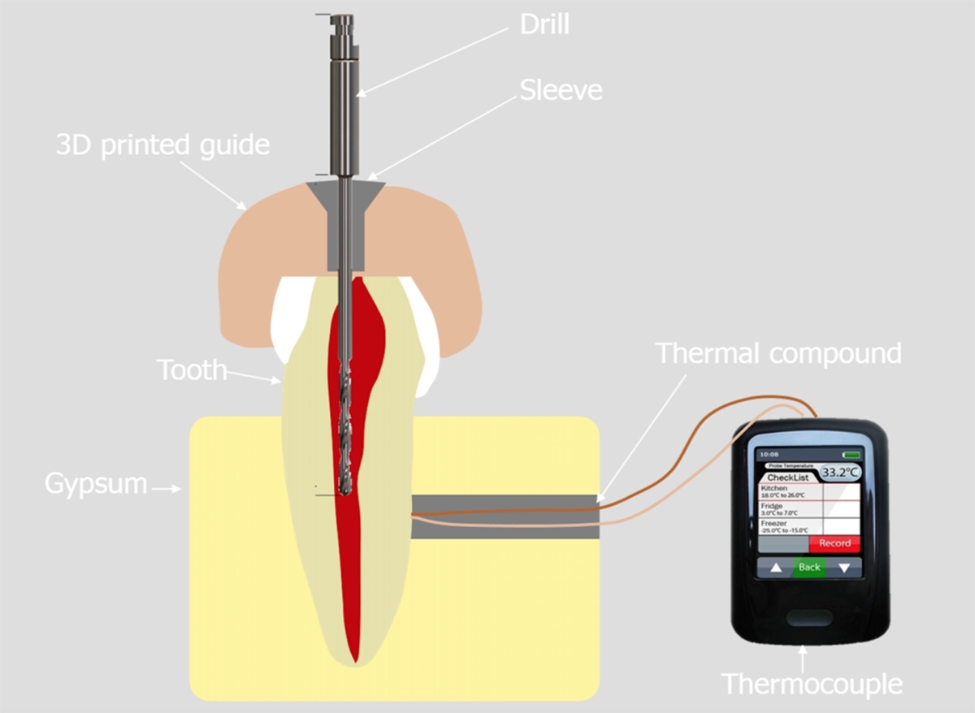
Setup for thermal measurement

The thermocouple channel in the support was filled with PK-Zero thermal compound (Prolimatech, Taiwan), and the thermocouple was fed into the channel up to the root surface. The other end of the thermocouple was connected to a digital thermometer (EL-EnviroPad-TC, Lascar Electronics Ltd., Salisbury, UK) (Fig. 5).

A marking on the tooth was made through the guide sleeve, enamel was removed for all teeth using a diamond bur and dentin was removed for certain sets of teeth, creating an access cavity (AC) (see group descriptions). Access cavities were prepared with the same sized round diamond burs parallel to the long axis of the tooth. Access cavity width was set by the diameter of this bur. Cavities were prepared until the pulp chamber was reached, or in the case of calcified pulp chambers, preparation was continued until the depth of the cementoenamel junction was reached.

### Drilling protocol

Endodontic preparation through the guide was performed by the same operator, with over five years of experience in guided implantology and endodontics (A.M.). The drill feed rate was standardized using a digital scale. The same micromotor (Bien-Air Chiropro 980, Bien-Air Surgery SA, Le Noirmont, Switzerland) with a 6:1 endodontic handpiece (VDW, München, Germany) was used for the preparation of all teeth.

### Study groups

Four parameters affecting temperature change were investigated in the study: (a) access cavity preparation prior to endodontic drilling, (b) drill speed, (c) cooling and (d) cooling fluid temperature. Twelve teeth were allocated into each of the following test groups:

Group 1:

Guided drilling without access cavity preparation (w/o AC) at 800 RPM without cooling (w/o C).

Group 2:

Guided drilling without access cavity preparation (w/o AC) at 1000 RPM without cooling (w/o C).

Group 3:

Access cavity (w/AC) preparation prior to endodontic drilling and guided drilling at 1000 RPM without cooling (w/o C).

Group 4:

Access cavity (w/AC) preparation prior to endodontic drilling, guided drilling at 800 RPM without cooling (w/o C).

Group 5:

Access cavity (w/AC) preparation prior to endodontic drilling, guided drilling at 1000 RPM speed, cooling (w/C) with a room temperature (21 °C) coolant.

Group 6:

Access cavity (w/AC) preparation prior to endodontic drilling, guided drilling at 1000 RPM speed, cooling (w/C) with a chilled (4–6 °C) coolant.

### Statistical analysis

Sample size was calculated in G*Power version 3.1.9.7. Considering 80% power, 5% alpha error and effect size of 0.5, a minimum of ten samples per group were required. Since the size of the support enabled the fit of more teeth, we analyzed twelve samples per group. This sample size was in accordance with previous studies regarding the subject [7]. The statistical analyses were performed with SPSS v. 25.0 (SPSS, Chicago, IL). The Kolmogorov‒Smirnov test was applied to test the normality of the distribution of the data. The changes in temperatures were compared between guided endodontic root canal preparation groups with one-way ANOVA, followed by Tukey’s HSD post hoc test. P values below 0.05 were considered significant.

## Results

No prior recommendation for drill speed in guided endodontic drilling was found among published scientific literature; therefore, we conducted a preliminary study to determine optimal drill speeds. In this preliminary experiment (data not shown) it was found rotary speeds of 1200 RPM and above did not improve drilling efficiency; however, rapid heating of the drill was observed and drill breakage often occurred. Therefore, 1000 RPM was chosen for the cooling efficiency test. On the other end of the spectrum, speeds below 800 RPM were associated with drastically reduced drilling efficiency and with a prolonged temperature rise, resulting in higher peak temperatures than speeds of 800 RPM and above.

Mean temperature elevations are shown in Table 1.

**Table 1:** Mean temperature elevations for each group

	RPM	Cooling	Trepanation	Number of teeth	Mean temperature elevation (°C)	Standard deviation
Group 1.	800	No	No	12	14.62 °C	0.63
Group 2.	1000	No	No	12	13.76 °C	1.24
Group 3.	1000	No	Yes	12	10.09 °C	1.32
Group 4.	800	No	Yes	12	8.90 °C	0.50
Group 5.	1000	Yes (21 °C)	Yes	12	4.01 °C	0.22
Group 6.	1000	Yes (4–6 °C)	Yes	12	1.60 °C	1.17

The highest mean temperatures were observed for drilling without prior access cavity preparation. In this setup, drill speeds of 800 RPM (Group 1.) resulted in higher mean temperatures (14.62 °C ± 0.63) than drill speeds of 1000 RPM (Group 2.) (13.76 °C ± 1.24). The difference between these two groups was not statistically significant (p = 0.243), however, both groups showed significantly higher (p < 0.01) temperatures than any of the access cavity groups (3.,4.,5.,6.)

In groups in which access cavity preparation was applied (Groups 3 and 4) significantly lower (p < 0.01) mean temperature values (10.09 °C ± 1.32 and 8.90 °C ± 0.50, respectively) were measured in comparison to the no access cavity groups (Groups 1 and 2). However, both groups 3 and 4 showed significantly higher mean temperatures than the groups in which cooling was used (Groups 5 and 6; p < 0.01). In this setup (access cavity prepared, no cooling applied), the drill speed had a significant effect, in which 1000 RPM resulted in significantly higher mean temperatures than when compared with 800 RPM (p < 0.05).

Cooling significantly decreased (p < 0.01) the mean temperature increase in both groups (5., 6.) (4.01 °C ± 0.22) and 6. (1.60 °C ± 1.17) compared to any of the uncooled groups (1., 2., 3., 4.). The temperature of the cooling liquid had a significant effect (p < 0.01), and the application of a chilled cooling liquid (Group 6.) proved more beneficial than using a room temperature liquid (Group 5.) at the same drill speeds (1000 RPM).

**Fig. 6:**
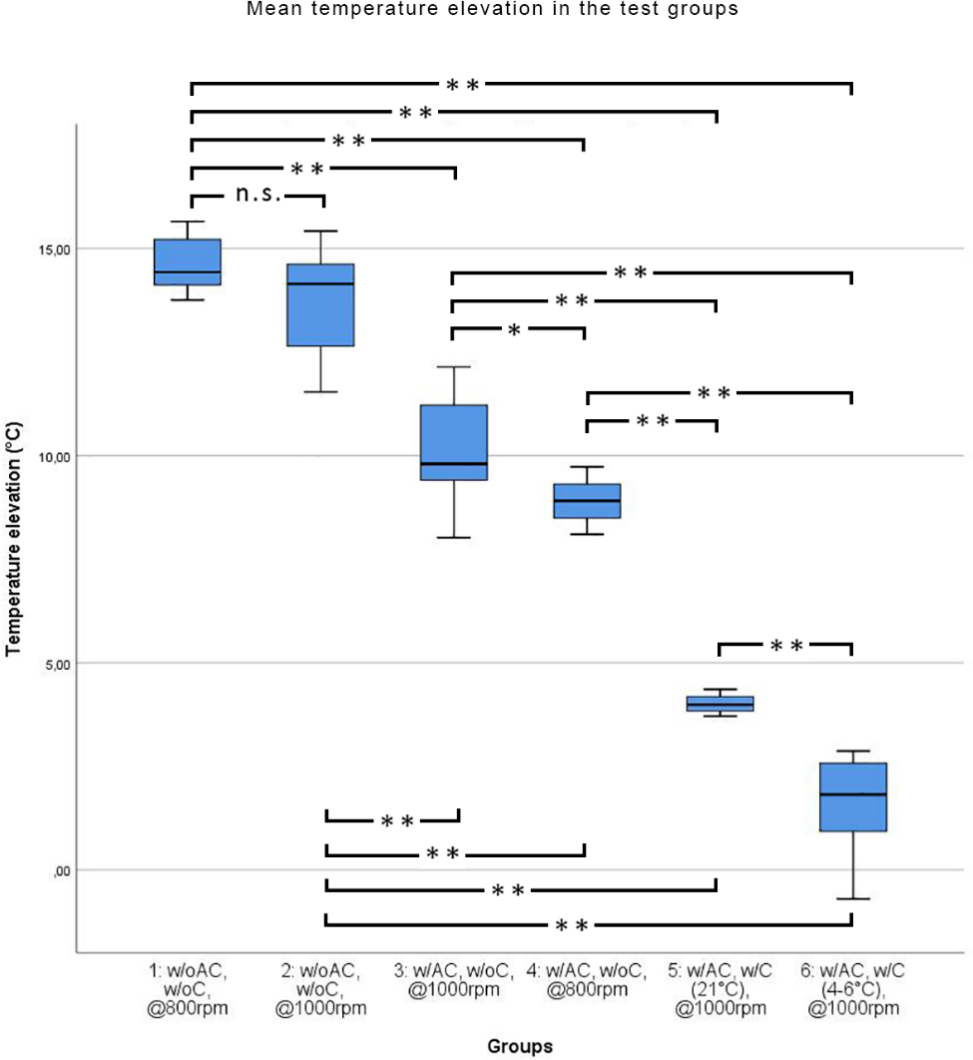
Temperature elevation in different groups (n.s.: not significant; *: p < 0.05; **: p < 0.01)

The results of the intergroup comparisons are shown in Fig. 6.

## Discussion

Guided root canal drilling leads to heat generation at the drill-dentine interface. Excessive heat generation may lead to collateral thermal damage of the tissues of the periodontal ligament surrounding the root [8]. According to Sauk et al. [9], hyperthermia at 43 °C can lead to decreased protein synthesis, thus altering the functions of periodontal ligament cells. Eriksson and Albrektsson [10] found 47 °C temperature for at least 1 min is necessary for bone damage visible by light microscopy. Kniha et al., in their systematic review, discovered a wide range of published threshold values and concluded, due to the heterogeneity of experimental setups, no exact temperature for bone necrosis can be determined [11]. Cunha et al. in their systematic review demonstrated how many factors may contribute to postoperative pain and discomfort in patients who underwent endodontic treatment [12]. It can be assumed temperature elevations even below the necrotic threshold values may also contribute to postoperative pain, therefore, any temperature elevation is to be avoided during endodontic treatments, if possible.

Most of the studies conducted on thermal bone damage are focused on direct heat transfer to the bone when examining critical temperatures. During guided endodontic drilling, heat is first transferred to the nonvital structure of dentine and only secondarily to bone. In this regard, preparation in the root canal is more similar to broken abutment screw removal from dental implants [13]. However, conclusions derived from these studies cannot be directly applied to guided endodontics for two main reasons. One premise implies titanium features better heat conductivity when compared with dentine, and the other premise is blood flow in the periodontal ligament has an attenuating effect upon heat transfer from the unvital structure to the bone.

Although various anatomical factors, including the length of the root, width of the remaining root canal and calcified tissue inside the root canal may contribute to heat generation, they are difficult to control. Procedural factors, such as the type of drill used, presence of a properly prepared access cavity, drill speed, cooling and temperature of the coolant may also contribute to heat generation. However, the importance and effect of these procedural factors have not yet been fully investigated in published scientific literature.

The results show all four tested drilling parameters affected heat generation during in vitro investigation.

The lack of access cavity preparation prior to guided endodontic drilling reportedly bears a detrimental effect, increasing root surface temperature by more than 10 °C regardless of the drilling speed applied.

Our data implies drilling speed also has a major effect on heat generation when the access cavity is prepared prior to guided drilling. Seemingly, a lower speed (800 RPM) results in less heat generation than higher speed (1000 RPM) drilling. The temperature values were also more consistent with lower speed preparations. This may indicate lower speed preparations are less sensitive to different root canal anatomies.

Additionally, cooling of the drill as well as the temperature of the cooling liquid have major effects on heat generation even when higher drill speeds were used. The highest measured temperature elevation with cooling was still lower than the lowest temperature elevation without cooling. In the two cases with the use of refrigerated cooling liquid, no temperature elevation was observed during the entire drilling process. Therefore, it can be assumed cooling the drill is the most predictable method to reduce collateral thermal damage.

The mean temperature data (4.01 °C ± 0.22) of Group 5 of our study (access cavity preparation followed by guided drilling at 1000 RPM and cooling with room temperature coolant) were consistent with the mean temperature data (5.07 °C) of the guided endodontic drilling group (access cavity preparation followed by drilling at 800 RPM for 120 s without cooling) from the study published by Zhang et al. [7]

It must be noted, these data only refer to the one specific drill type used for this study. Bur material, diameter, shape and blade configuration may also contribute to accuracy and heat generation; however, investigation of these parameters was beyond the scope of our study [14, 15].

## Conclusion

There is a growing need for the development of technical recommendations and protocols as the technique of guided root canal drilling becomes increasingly more accessible to dental practitioners. With the cautious evaluation of unswayed anatomical factors of the tooth and with the thorough understanding of influential procedural factors, the risk of collateral thermal damage during guided endodontic drilling can be minimized. Based on the results of our study, guided endodontic drilling at drill speeds not exceeding 1000 RPM following access cavity preparation, with constant cooling using a fluid cooler than room temperature, provides the best results in avoiding collateral thermal damage.

## Data Availability

The datasets used and/or analyzed during the current in vitro study are available from the corresponding author on reasonable request.
